# Injectable and In Situ Phospholipid-Based Phase Separation Gel for Sustained Delivery of Altrenogest

**DOI:** 10.3390/gels10120847

**Published:** 2024-12-23

**Authors:** Dongbo Li, Awn Abbas, Nanxin Li, Chao Li, Xiaoyang Ai, Lian Chen, Dongmei Dai, Gang Shu, Juchun Lin, Wei Zhang, Guangneng Peng, Haohuan Li, Funeng Xu, Hualin Fu

**Affiliations:** Department of Pharmacy, College of Veterinary Medicine, Sichuan Agricultural University, Chengdu 611130, China; 18081626755@163.com (D.L.); awni1115@gmail.com (A.A.); linanxinao1997@gmail.com (N.L.); 13835372941@163.com (C.L.); a1600063002@163.com (X.A.); 15908499457@163.com (L.C.); 18884363072@163.com (D.D.); dyysg2005@sicau.edu.cn (G.S.); juchunlin@126.com (J.L.); zhangwei26510c@126.com (W.Z.); pgn.sicau@163.com (G.P.); lihaohuan7@163.com (H.L.); funengxu@sicau.edu.cn (F.X.)

**Keywords:** altrenogest, gels, viscosity change, sustained release, pharmacokinetic

## Abstract

Altrenogest is a key regulatory hormone for intensive and batch management of reserve sows in breeding farms. As a synthetic hormone, altrenogest could make ovaries stay at the initial stage of follicles and inhibit estrus and ovulation in animals. However, the currently used oral altrenogest solution needs to be administered continuously every day for more than two weeks in clinical practice. In this study we developed a phospholipid-based injectable gel carrying altrenogest to decrease the number of administrations, sustain release of the drug, and enhance therapeutic efficacy for clinical use. The altrenogest gel had a viscosity of 100 cP before phase transition and over 1,000,000 cP after phase transition. In vitro, altrenogest can be continuously released from gel for over two weeks. The pharmacokinetic results showed that the AUC _(0–∞)_ of the altrenogest gel was almost double that of the altrenogest solution. The MRT _(0–∞)_ was 40.92 ± 7.21 h and the t_1/2_ of the altrenogest gel was 80.03 ± 20.79 h. The altrenogest gel demonstrated excellent fluidity, ease of injectability, high drug-loading capacity, and appropriate sustained-release characteristics both in vitro and in vivo, making it a potential drug delivery system for swine production.

## 1. Introduction

Altrenogest is a key regulatory hormone for intensive and batch management of reserve sows in breeding farms, which increases the production capacity of sows, reduces the biosafety risk of farms and improves the economic benefits of farms, and it has been widely used worldwide [1,2]. As a synthetic hormone, altrenogest has a similar mode of action to that of natural progesterone. It can make the ovaries stay at the initial stage of follicles and inhibit estrus and ovulation in animals [3]. After altrenogest withdrawal, the follicles developed and matured again, achieving the effect of synchronized estrus in the treated animals [4].

Altrenogest minimizes the variability in gestation duration and synchronizes the initiation of parturition in sows [5], while enhancing the uterine environment during the peri-implantation phase in pigs without adversely affecting corpora lutea development [6]. Studies have shown that initiating a synchronous estrus program regulated by altrenogest during the luteal phase of sows can induce a better environment for follicular development and ovulation in gilts [7]. Sows using altrenogest in the late lactation period can have a shorter weaning-to-estrus cycle and larger follicles during estrus, which is conducive to synchronous pregnancy [8]. Studies also have shown that altrenogest can improve the fertility of sows and reduce the impact of environmental temperature on reproduction [9].

The primary clinically utilized dosage form of altrenogest is an oral solution [10], which can be administered by spraying it onto feed or directly into sows using a dosing gun. Some scholars have developed allyl progesterone soft capsules, which can more accurately control the dosage of medication [11]. Studies have shown that chelation with HP-β-CD is an efficient method for improving altrenogest solubility and oral bioavailability [12]. However, it is necessary to ensure that altrenogest has efficacy for 14–18 days in clinical use, which leads to the continuous daily administration of the oral preparations, with great labor time costs to farms.

The gel delivery system has been extensively studied and utilized in the past decade due to its excellent sustained-release abilities [13,14]. A chitosan/sodium alginate hydrogel utilized as a carrier for the formulation of a sustained-release gel can continuously release up to 90% of berberine over a period of 24 days [15]. Some scholars have developed an effective drug delivery system of fibroin/chitosan thiourea gel that shows the sustained long-term release of drugs from the gels for more than 20 days [16]. Studies have shown that sustained in vitro release of drugs from methacrylated chondroitin sulfate gel was observed over a 24-day period [17]. Polyvinyl alcohol-based gel may offer an efficient, regulated, and secure sustained drug administration in both in vitro and in vivo analysis for up to 32 days [18].

These gel delivery systems can continuously release drugs for a long time, which could help the clinical use of altrenogest. Recently, some scholars have proposed a phospholipid based sustained-release gel, which can form gel in situ in the body and continuously release drugs to treat arthritis [19]. The research shows that this phospholipid-based gel can continuously release chlorogenic acid in vivo for 15 days [20]. In this research, an injectable gel loaded with altrenogest was produced for the first time to reduce administration frequency, ensure prolonged drug release and enhance drug efficacy for clinical application. Altrenogest can form complexes with phospholipids, facilitating the creation of altrenogest gel with a high drug-loading capacity to enhance sustained release efficiency. Upon injection into the vivo, the sol–gel transition occurred as ethanol diffused out of the gel and water diffused into it. The low water solubility of phospholipids impeded the fast release of altrenogest due to precipitation. This study examined the morphology, viscosity, release profile and storage stability in vitro. The pharmacokinetic properties and sustained-release impact were further examined in vivo. These investigations have established the groundwork for clinical application.

## 2. Results and Discussion

### 2.1. Characterization and Viscosity of Blank Gel

**Figure 1:**
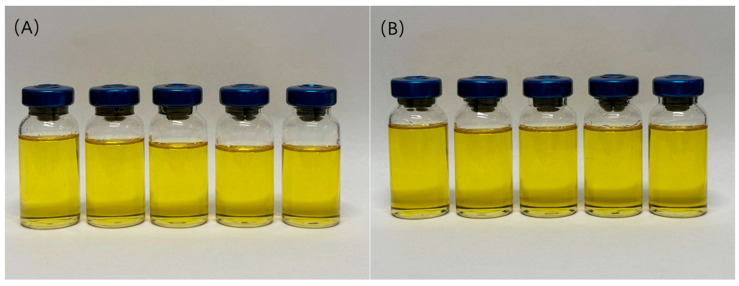
(**A**) From left to right are formulations 1–5. (**B**) From left to right are formulations 6–10.

**Figure 2:**
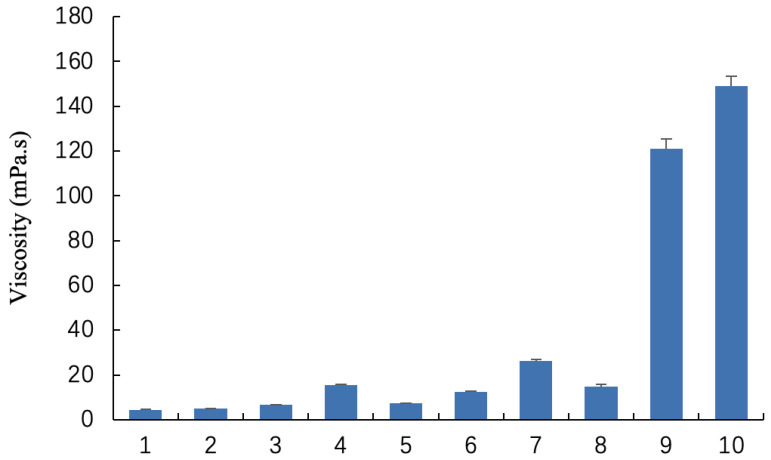
Viscosity of blank gels formulations 1–10.

Blank gels with different formulations were prepared according to Table 1. As shown in Figure 1A,B, the blank gels before phase transition were clear and transparent yellow solutions in appearance, indicating that the soybean phospholipid completely dissolved in caprylic/capric triglyceride and ethanol. With the increase in soybean phospholipid in the formulation, the viscosity of the gel increased in a positive proportion. Meanwhile, the addition of caprylic/capric triglyceride in the prescription also increased the viscosity of the gel, as shown in Figure 2.

The application of soybean phospholipid as a matrix skeleton in drug delivery systems has been reported. Novel phospholipid-complexed nanofibrous scaffolds were fabricated [21]. Curcumin-loaded liposomes prepared from soybean phospholipids displayed superior anti-inflammatory effects [22] and good stability [23]. Studies have also shown that soybean phospholipid is a promising steric stabilizer in nanosuspension drug delivery systems [24]. Caprylic/capric triglyceride is a medium chain triglyceride; studies have shown that the addition of caprylic/capric triglyceride can significantly reduce the sizes of nanocapsules and improve the size distribution of the nanocapsules [25]. In this study, different proportions of soybean phospholipids and caprylic/capric triglycerides were dissolved in ethanol to form a stable blank gel.

### 2.2. Effect of Physiological Saline on the Viscosity of Blank Gel

**Figure 3:**
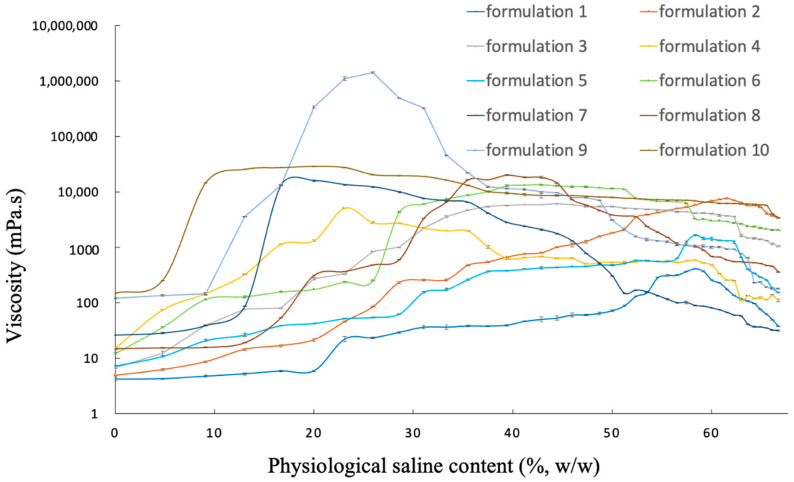
Viscosity changes of gels containing different physiological saline contents.

For injectable original gels, low initial viscosity was required for easy injection; in situ implants with high viscosity were required to control drug release after injection [26]. The viscosity of blank gels contained various amounts of physiological saline to predict their phase transition behavior. The proportion of all prescriptions is shown in Table 1. The viscosity of all gels rose with increasing physiological saline content in a certain range (Figure 3). Through the results of formulations 2, 6 and 9, we found that with more phospholipid content in the prescription, the viscosity could be improved. When we compared formulations 1, 2, 3 and 4, formulations 5, 6 and 7 and formulations 8 and 9, respectively, we could find that the addition of caprylic/capric triglyceride in the prescription improved the viscosity to reach the condition of forming a high-viscosity in situ drug release library.

Similar studies have shown that the addition of medium chain triglycerides to the phospholipid-based gel can improve the gel viscosity and further form a drug repository [27]. The sharp viscosity increase at the beginning was required for rapid phase transition [28], which was beneficial to controlling the initial burst drug release, and the further increase with increased physiological saline content was important for maintaining sustained drug release [29]. Overall, formulation 9 had a viscosity of 100 cP before phase transition, which was consistent with easy injection, and a viscosity over 1,000,000 cP after phase transition in contact with physiological saline. It also conformed to the long-term slow release of the drug as a release reservoir.

When the gel was in contact with physiological saline, the soybean phospholipid would quickly form precipitation and increase the viscosity of the gel. Most of the phospholipid in the formulation had been precipitated; adding normal saline would dilute the viscosity of the gel. It was assumed that the dilution effect of physiological saline contributes to the decrease in viscosity at high physiological saline content. When physiological saline content increases to a certain value, where the dilution effect of physiological saline is greater than the effect of the phospholipids’ precipitate formation, the viscosity of gel systems start to decrease. There was no doubt that the insolubility of phospholipids in an aqueous solution was the main reason for this viscosity change. By adding an appropriate amount of normal saline into the gel system, its viscosity increased exponentially, indicating its great ability to form an implant upon exposure to an aqueous solution.

### 2.3. Characterization of Altrenogest Gel

**Figure 4:**
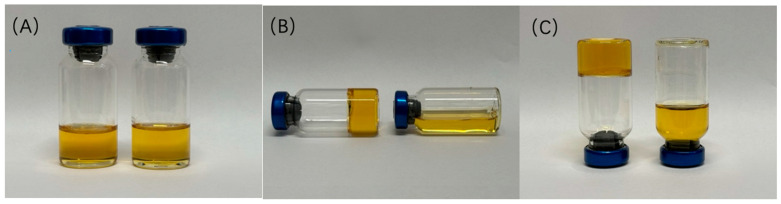
(**A**–**C**) Photographs of altrenoest gel before (**right**) and after (**left**) phase transition in vitro.

**Figure 5:**
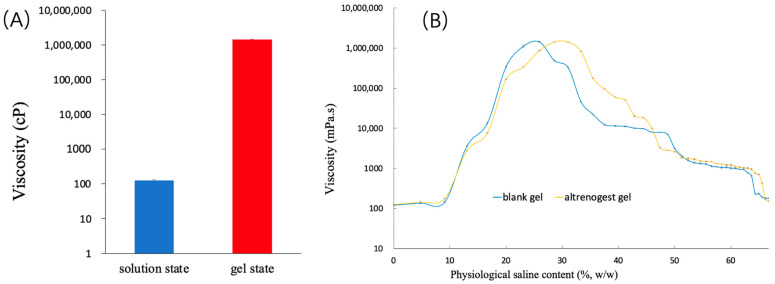
(**A**) The viscosity of altrenogest gel in sol and gel states at 25 °C (n = 3). (**B**) Viscosity changes of blank gel and altrenoest gel containing different physiological saline contents.

As shown in Figure 4, altrenogest gel was transformed from a transparent solution into a gel after the occurrence of a phase transition. The viscosity of the altrenogest gel state was significantly higher than that of the initial solution state (Figure 5A). The phase transition process of blank gel and altrenogest-loaded gel was basically the same (Figure 5B). The physiological saline content with maximum viscosity of the altrenogest gel appeared at 30% of normal saline; this was slightly higher than that of blank gel, due to the addition of altrenogest into the system. The results showed that the phase transition process of the blank gel was not affected by the addition of altrenogest, and the gel system could be used as a long-term drug release library. Altrenogest was loaded into gels to promote rapid gel formation in situ via a solution–gel transformation mechanism, thereby achieving a sustained, controlled release. Furthermore, altrenogest was distributed in the oil–water interface layer and within the oil phase. Solvent exchange drives phase transitions, and phospholipid vesicle formation and rupture are likely to promote drug release and gel degradation [30].

The development and application of PLGA hydrogel for continuous delivery of therapeutic agents is the hottest research direction for gels. Due to its biocompatibility, biodegradability and other multifunctional properties, it has been widely used in biomedical research [31]. For PLGA-based gel, drugs need to be wrapped in gel by the physical embedding method or chemical bonding method. For traditional hydrogel systems, most of them carry water-soluble drugs, and for liposoluble drugs, the phospholipid-based gel system has more advantages. The drug can be directly dissolved in caprylic/capric triglyceride or ethanol. Similarly, phospholipids have good biocompatibility and biodegradability and can be safely used in clinical practice. The research has shown that PLGA hydrogel loaded with water-soluble drugs can form in situ gel to achieve a sustained-release effect after injection [32].

The gelling temperature of gel based on poloxamer studied by some scholars is 29 °C, and the gelling time is 5 min [33]. Some studies have also shown that the temperature sensitivity of PLGA hydrogel can be changed by regulating PLGA monomer [34]. Different from the traditional hydrogel and PLGA hydrogel, the phospholipid-based gel has no fixed gelling temperature. The condition for forming the gel of phospholipid-based gel is that after the exchange of ethanol and external water, the phospholipid precipitates rapidly and forms a drug storage warehouse in situ.

### 2.4. In Vitro Release Study

**Figure 6:**
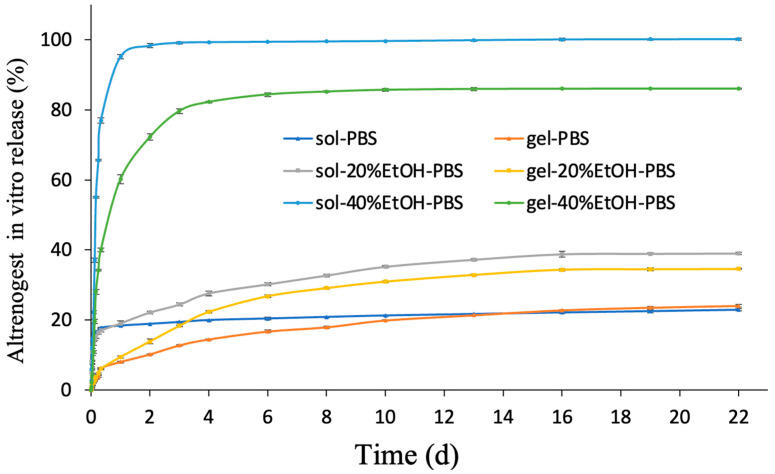
In vitro release of altrenogest from solution and gel in various release mediums.

Altrenogest released slowly in PBS medium. We also used PBS with 20% and 40% ethanol as the release medium. Either 20% ethanol or 40% ethanol was added in PBS (*v*/*v*) to stimulate the erosion of the altrenogest gel and altrenogest solution. Figure 6 shows the cumulative release profile. For the altrenogest solution group, the release of altrenogest was fast in 40% ethanol-containing mediums with 100% altrenogest release in 2 d. The final release rate of the altrenogest solution group was about 40% in 20% ethanol medium and 20% in PBS medium, respectively. In contrast, the release of altrenogest gel was significantly slower. In PBS medium, only 20% of altrenogest was released from the gel. The 30% altrenogest was released for 16 days in the release medium of 20% ethanol. In the release medium containing 40% ethanol, about 80% of altrenogest was released from gel. These results suggest that altrenogest gel effectively blocks altrenogest release due to the insoluble nature of soybean phospholipids and caprylic/capric triglycerides in the aqueous environment.

Cumulative release of altrenogest in in vitro experiments increased with ethanol concentration in the release medium, which may reflect the fact that both excipients, phospholipids and altrenogest, are soluble in ethanol. Thus, increasing the ethanol content in the release medium may facilitate diffusion of phospholipids and altrenogest out of the gel matrix, accelerating matrix degradation and drug release [35]. Meanwhile, another study has shown that the gel based on phospholipids can release the drug for more than two weeks. PBS in the release medium makes the gel phase change to form a drug warehouse, but ethanol will slowly corrode the phospholipid and release the drug [36].

### 2.5. Storage Stability

**Figure 7:**
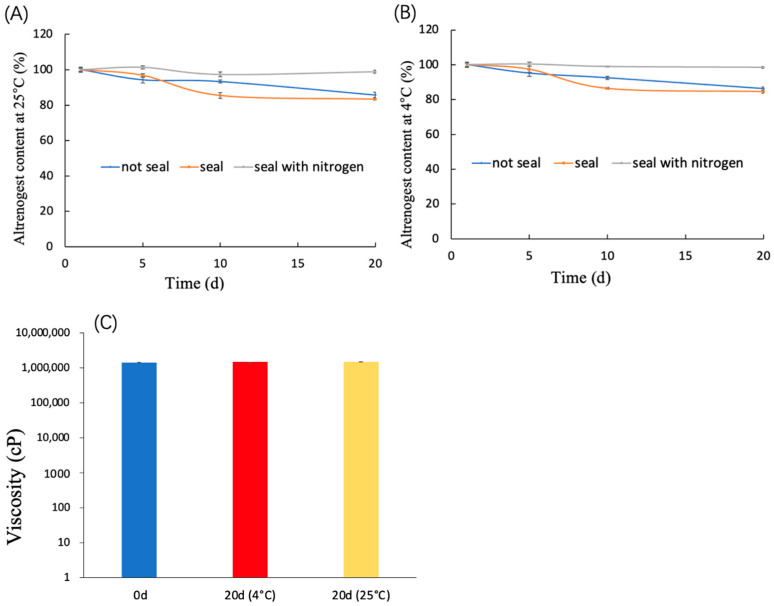
(**A**) Altrenogest content at different storage times in 25 °C. (**B**) Altrenogest content at different storage times in 4 °C. (**C**) Viscosity before (0 d) and after (20 d) storage.

The storage stability of altrenogest gel was evaluated by altrenogest content and viscosity. The content of altrenogest in the gel would change significantly under different storage conditions, as shown in Figure 7A,B. There was no change in the content of altrenogest in the sealed nitrogen-filled condition for 20 days at 4 or 25 °C. Once the altrenogest gel was exposed to air, even if it was sealed in a vial and exposed to a small amount of air, the content of altrenogest would decline to about 80% in 20 days, even at 4 or 25 °C. The soybean phospholipid had a purity over 90% of phosphatidylcholine, and its storage stability was mainly related to oxidation [37]. According to the experimental results, when stored in a sealed bottle filled with nitrogen, its stability can be effectively extended. As shown in Figure 7C, the viscosity of altrenogest gel did not change significantly after 20 days, whether at 4 or 25 °C.

### 2.6. Pharmacokinetic Study

**Figure 8:**
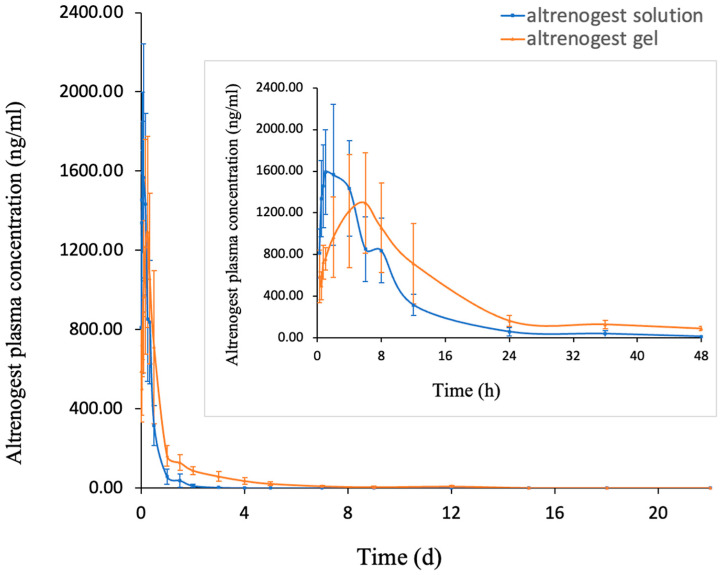
Pharmacokinetic profiles of altrenogest solution (200 mg/kg) and altrenogest gel (200 mg/kg).

Scholars have established a detection method for altrenogest in horses [38]; the pharmacokinetics of altrenogest in rats have not been reported. Our study shows a new method for detecting altrenogest in rat plasma by LC-MS. In our study, the blood concentration–time profiles of altrenogest sol after oral administration and altrenogest gel after hypodermic injection in rats are shown in Figure 8. Compared with the altrenogest sol group, the altrenogest gel group absorbed slowly and had a lower absorption peak. Table 2 illustrates the corresponding pharmacokinetic parameters. The AUC _(0–∞)_ of the altrenogest gel group was almost double that of the altrenogest sol group, and the MRT _(0–∞)_ increased from about 8 h to 40 h. The altrenogest sol had a short plasma elimination half-life (t_1/2_) of 8.51 ± 2.38 h, whereas the t_1/2_ of the altrenogest gel was 80.03 ± 20.79 h. The altrenogest sol showed T_max_ in about 2 h, and the high maximum blood concentration (C_max_) of altrenogest was up to 1626.46 ± 421.81 μg/L. In contrast, the altrenogest gel kept a slower and steadier release of altrenogest, with the C_max_ decreasing to 1433.49 ± 416.41 μg/L.

The main pharmacokinetic parameters of two different oral solutions of altrenogest administered daily on the market were basically the same: t_1/2_ was 3.63 ± 0.72 h and 3.45 ± 0.63 h [39]. They were far less than the half-life of altrenogest gel, 80.03 ± 20.79 h, which fully demonstrates the sustained-release effect of altrenogest gel in vivo. We speculated that after the subcutaneous injection of altrenogest gel, the ethanol was absorbed quickly, while taking away part of the altrenogest, and the remaining altrenogest and phospholipid would form a drug storage warehouse in situ. Due to the biocompatibility of phospholipids, phospholipids are slowly absorbed by the body, while altrenogest is also slowly absorbed to achieve the characteristics of slow drug release. The drug storage warehouse based on phospholipids would be completely absorbed for a long time, and altrenogest is also completely absorbed slowly. This leads to an extension of the half-life of altrenogest. We guess that the pharmacokinetics of altrenogest in clinical pigs might have a longer sustained-release effect, as the single dose in pigs is higher than rats, and the drug reservoir formed by phospholipids may be larger, requiring a longer time for corrosion and absorption. The preparation can continuously release altrenogest in the body for 14 days, which could replace the current oral altrenogest solution on the market. Meanwhile, other scholars who gave altrenogest to gilts followed the clinical practice for 18 consecutive days [40]. Plasma concentrations of altrenogest had a certain degree of fluctuation, without significant accumulations. There is no need to worry about the accumulation of drugs in the pigs.

## 3. Conclusions

In this study, we prepared altrenogest gel with a soybean phospholipid and caprylic/capric triglyceride in order to achieve sustained release and efficient delivery of altrenogest for intensive and batch management of reserve sows. Altrenogest gel was prepared with a simple method, which is easy for scaling up and commercialization. The injectable altrenogest gel showed a proper sustained-release behavior in vitro and in vivo, which is a promising sustained-release formulation for pig farming. At present, pharmacokinetic and pharmacodynamic studies have not been conducted on sows, and it is also necessary to evaluate the irritation and safety of the injectable gel. In the future, we will further study the drug-release and sustained-release mechanisms of altrenogest gel and convert it into a new veterinary drug preparation clinically. If altrenogest gel is marketed as a new veterinary drug preparation, it can fill the gap left by altrenogest not having a long-term sustained release preparation, reduce the labor time cost, increase the breeding efficiency and promote the development of batch management of sow production in intensive pig farms.

## 4. Materials and Methods

### 4.1. Materials

Altrenogest and Caprylic/capric triglyceride were obtained from Ningbo Sansheng Biological Technology Co., Ltd. (Ningbo, China). Soybean phospholipid was provided by Shenyang Tianfeng biological Pharmaceutical Co., Ltd. (Shenyang, China). Ethanol was purchased from Chengdu Chron Chemicals Co., Ltd. (Chengdu, China).

### 4.2. Preparation of Blank Gel and Altrenogest Gel

The blank gel was prepared with soybean phospholipid, caprylic/capric triglyceride and ethanol in a ratio of 70:15:15 (*w*/*w*/*w*) (or other ratios in Table 1). They were mixed under magnetic stirring at room temperature for 2 h, resulting in a homogenous liquid system after soybean phospholipid dissolved completely.

To prepare altrenogest gel, altrenogest was added into blank gel and stirred for 30 min to acquire a homogeneous drug delivery system at solution state. The altrenogest concentration in gel was 40 mg/mL.

### 4.3. Viscosity Measurement

The viscosity of gels was assessed utilizing a Rotational Viscometer (NDJ-5S, Shanghai Changji Geological Instrument Co., Ltd., Shanghai, China). A certain volume of gel was introduced into a container that corresponded in size to the utilized rotator. The added gel had to completely submerge the rotator. Prior to measurement, the rotational speed was established to guarantee the torque value remained within the range of 10–90%. The viscosity measurement was recorded only once all values displayed on the screen stabilized.

### 4.4. Effect of Physiological Saline on Viscosity of Gel

The gels were combined with varying quantities of physiological saline (0.01 M) by stirring for a minimum of 10 min at an ambient temperature. The physiological saline concentration in the final mixture ranged from 0 to 66.7% (*w*/*w*). The viscosity of the resulting homogeneous mixture was assessed as previously described. The result was illustrated as a viscosity–physiological saline content curve.

### 4.5. Phase Transition of Altrenogest Gel

Altrenogest gel was introduced into water to characterize the gel transition of the system upon exposure to water and ethanol diffusion. The appearance of the systems before and after the phase transition was documented following the complete mixing of altrenogest gel with 30% (*w*/*w*) physiological saline (0.01 M).

### 4.6. Sustained Drug Release Behavior

The altrenogest gel or altrenogest solution was incorporated into the dialysis bags, respectively, which had a molecular weight cut-off of 8–14 kD. Upon securely wrapping the two ends, the dialysis bag containing the sample was immersed in 5 mL of release media (PBS or PBS with ethanol, pH 7.4) and thereafter agitated in a horizontal shaker at 100 rpm at 37 °C. At specified time intervals, the medium external to the dialysis bag was extracted and substituted with 5 mL of fresh medium. The collected medium was diluted to 5 mL, and the altrenogest content was quantified by high ferformance liquid chromatography (HPLC, Waters 2487, Waters Corporation, Milford, MA, USA).

### 4.7. Storage Stability Study of Altrenogest Gel

After the altrenogest gel was divided into different sample bottles, nitrogen was injected into the upper part to avoid oxidation. They were divided into three groups. The first group was not sealed in the sample bottle, the second group was sealed and the third group was sealed with nitrogen filled vials. The bottles were put at 25 °C and 4 °C. At pre-determined time intervals (0 d, 5 d, 10 d, 20 d), the altrenogest gel was taken out to detect the content by HPLC. Take the content on day 0 as the initial content, set 100%, and calculate the percentage of remaining altrenogest at each time point. At the same time, the altrenogest gel was stored at 4 °C and 25 °C with nitrogen, and the viscosity of the gel was measured again 20 days later.

### 4.8. In Vivo Pharmacokinetic Study

Female Sprague Dawley rats (190–210 g) were randomly divided into the altrenogest sol group and altrenogest gel group. The altrenogest sol group was administered by gavage with altrenogest suspension solution (200 mg/kg) and the altrenogest gel group was administered by hypodermic injection with altrenogest gel (200 mg/kg). Blood samples (300 μL) were collected from the eyes at specific time points (15 min, 30 min, 60 min, 2 h, 4 h, 6 h, 8 h, 12 h, 24 h, 36 h, 2 d, 3 d, 4 d, 5 d, 7 d, 9 d,12 d, 15 d, 18 d, 22 d) after administration and then placed into heparinized tubes.

The samples were centrifuged immediately at 5000 rpm for 10 min to collect 50 μL plasma. The concentration of altrenogest in the plasma was monitored by liquid chromatography–mass spectrometry (LC-MS/MS, Agilent 8050, Agilent Technologies Co., Ltd., Santa Clara, CA, USA). Briefly, 50 μL of plasma sample was spiked with 50 μL levonorgestrel solution (internal standard) and extracted with 200 μL of acetonitrile by vortexing for 5 min and centrifugation at 10,000 rpm for 10 min. Next, the supernatant was filtered through 0.22 μm organic filter membrane before analysis. Data processing and analysis were carried out in DAS software, 2.0 version.

### 4.9. Statistical Analysis

All data were represented as mean ± standard deviation (SD). The t-test was employed to evaluate comparisons between the two groups. One-way ANOVA was used to evaluate comparisons between several groups. A *p*-value below 0.05 was deemed statistically significant.

## Figures and Tables

**Table 1:** Different ratios of blank gels (%, *w*/*w*/*w*).

Blank Gels	Soybean Phospholipid	Caprylic/Capric Triglyceride	Ethanol
Formulation 1	40	0	60
Formulation 2	40	15	45
Formulation 3	40	30	30
Formulation 4	40	45	15
Formulation 5	55	0	45
Formulation 6	55	15	30
Formulation 7	55	30	15
Formulation 8	70	0	30
Formulation 9	70	15	15
Formulation 10	85	0	15

**Table 2:** In vivo pharmacokinetic parameters. Data are presented as mean ± SD (*n* = 6).

Group	AUC _(0–∞)_ (μg/L·h)	MRT _(0–∞)_ (h)	t_1/2_ (h)	T_max_ (h)	C_max_ (μg/L)
Altrenogest sol	14,963.70 ± 1891.01	8.43 ± 1.69	8.51 ± 2.38	2.00 ± 1.22	1626.46 ± 421.81
Altrenogest gel	26,560.91 ± 6013.48 *	40.92 ± 7.21 *	80.03 ± 20.79 *	5.33 ± 2.00 *	1433.49 ± 416.41

Note: C_max_ represents maximum plasma concentration, t_1/2_ represents plasma elimination half-life, T_max_ represents time to maximum plasma concentration, AUC _(0–∞)_ represents area under the curve, MRT _(0–∞)_ represents mean residence time. Compared to altrenogest sol, * means *p* < 0.01.

## Data Availability

The original contributions presented in the study are included in the article. Further inquiries can be directed to the corresponding author.
